# Oral magnesium supplementation for insomnia in older adults: a Systematic Review & Meta-Analysis

**DOI:** 10.1186/s12906-021-03297-z

**Published:** 2021-04-17

**Authors:** Jasmine Mah, Tyler Pitre

**Affiliations:** 1 Department of Medicine, Dalhousie University, Halifax, NS Canada; 2 Division of Internal Medicine, McMaster University, Hamilton, ON Canada; 3 Michael G. DeGroote School of Medicine (Waterloo Regional Campus), McMaster University, Hamilton, ON Canada

**Keywords:** Magnesium, Supplementation, Sleep, Insomnia, Geriatrics

## Abstract

**Background:**

Magnesium supplementation is often purported to improve sleep; however, as both an over-the-counter sleep aid and a complementary and alternative medicine, there is limited evidence to support this assertion. The aim was to assess the effectiveness and safety of magnesium supplementation for older adults with insomnia.

**Methods:**

A search was conducted in MEDLINE, EMBASE, Allied and Complementary Medicine, clinicaltrials.gov and two grey literature databases comparing magnesium supplementation to placebo or no treatment. Outcomes were sleep quality, quantity, and adverse events. Risk of bias and quality of evidence assessments were carried out using the RoB 2.0 and Grading of Recommendations Assessment, Development and Evaluation (GRADE) approaches. Data was pooled and treatment effects were quantified using mean differences. For remaining outcomes, a modified effects direction plot was used for data synthesis.

**Results:**

Three randomized control trials (RCT) were identified comparing oral magnesium to placebo in 151 older adults in three countries. Pooled analysis showed that post-intervention sleep onset latency time was 17.36 min less after magnesium supplementation compared to placebo (95% CI − 27.27 to − 7.44, *p* = 0.0006). Total sleep time improved by 16.06 min in the magnesium supplementation group but was statistically insignificant. All trials were at moderate-to-high risk of bias and outcomes were supported by low to very low quality of evidence.

**Conclusion:**

This review confirms that the quality of literature is substandard for physicians to make well-informed recommendations on usage of oral magnesium for older adults with insomnia. However, given that oral magnesium is very cheap and widely available, RCT evidence may support oral magnesium supplements (less than 1 g quantities given up to three times a day) for insomnia symptoms.

**Supplementary Information:**

The online version contains supplementary material available at 10.1186/s12906-021-03297-z.

## Introduction

### Description of the condition

Insomnia is an increasingly common medical condition reported by up to 50% of older adults, defined as individuals greater or equal to 55 years old [1, 2]. A diagnosis of insomnia according to the International Classification of Sleep Disorders, involves difficulties initiating sleep, difficulties with sleep maintenance and subsequent daytime impairment in function [3].

### How the intervention may work

Magnesium (Mg) supplementation is often purported to improve sleep; however, as both an over-the-counter (OTC) sleep aid and a complementary and alternative medicine (CAM), there is limited evidence to support this assertion [4, 5]. Hence, no previous reviews on this topic were identified (see Additional File 1).

**Fig. 1:**
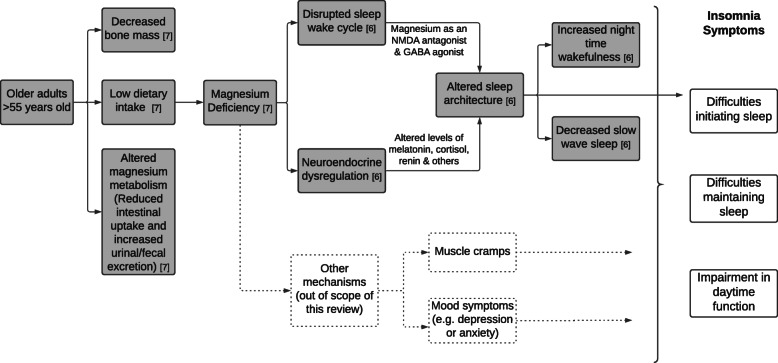
Possible biological mechanism & logic model underlying how magnesium supplementation influences older adult insomnia symptoms

The biological mechanisms linking magnesium to sleep are not well understood. A possible explanation summarising previous literature, is depicted in Fig. 1 [6, 7]; this also serves as a logic model guiding the research question and outcomes examined in this review. In brief, older adults are at higher risk of magnesium deficiency leading to altered sleep architecture though neuroendocrine dysregulation or disrupted sleep-wake cycles leading to the symptoms of insomnia.

### Importance of this review

Insomnia is linked to a high health and economic burden. Insomnia in older adults is correlated with compromised memory, cognitive impairment and dementia, increased falls, and poorer quality of life [1]. The annual economic cost to an older adult with insomnia is USD 1143 greater than matched controls without insomnia [8]. Magnesium is alluring because it is natural, inexpensive, widely available, and potentially beneficial as an OTC sleep aid; however, its clinical effectiveness and safety must be investigated to avoid placing patients at risk [4, 9]. The objective of this systematic review (SR) aims to assess the effects and safety of oral magnesium supplementation for older adults with insomnia.

## Methods

### Research question

Is magnesium supplementation taken by mouth, in comparison to placebo or no treatment, effective and safe for use by older adults with insomnia?

### Search and selection strategy

A SR of primary studies was conducted in MEDLINE (ALL segment from 1946 to October Week 12,020), EMBASE (1947 to October 16, 2020), Allied and Complementary Medicine (AMED) (1985 to October 2020), clinicaltrials.gov, OpenGrey and New York Academy of Medicine’s Grey Literature Report up to 18 October 2020. The electronic search was developed in MEDLINE, then translated to the remaining databases (chosen for their recognized pharmacological and CAM focuses). Two additional databases, PROSPERO and Cochrane Database of Systematic Reviews (CDSR) were hand-searched using the key term “magnesium”; zero reviews were found relevant to the research question. More details of search terms, rationale for inclusion of each database and exported strategies are available in Additional Files 1 and 2.

The search strategy used both key text words and indexed Medical Subject Headings (MESH) or Emtree terms. To balance comprehensiveness and relevance in this relatively unstudied topic, three concepts were identified: insomnia, older age and magnesium. A broad set of search terms was gathered for the first two concepts and then cross-referenced with existing systematic reviews on similar topics [10, 11]. Magnesium supplementation was identified to be the most specific concept; to increase the sensitivity of the search, the general term magnesium was used (instead of magnesium supplementation or intake). The key words and MESH terms were combined with OR Boolean operators and the three concepts were combined using the AND Boolean operator. Finally, a validated randomized control trial (RCT) search filter was applied [12]. Additionally, reference lists of included papers were hand-searched for records.

Citations were imported into a reference manager, EndNote (2018) [13], where duplicates were removed. The remaining records were uploaded to Covidence (2019) [14], a SR web platform where both authors independently screened titles and abstracts before proceeding to conduct independent full text retrievals and eligibility assessments.

### Eligibility criteria

Bibliographic records screened were eligible for inclusion if:

- Studies were RCTs of parallel-group or cross-over design. (Type of Study)
- The studies were conducted in a population of older adults where the majority (> 50% of participants) were greater or equal to 55 years old. (Population)
- Participants were diagnosed with insomnia by standardized measure (e.g. validated questionnaire), clinician evaluation/test (e.g. sleep laboratory), or self-reported sleep diary. (Population)
- Studies evaluated oral magnesium supplementation of any dose, frequency, duration or formulation in comparison to placebo or no treatment. (Intervention & Comparison)
- Outcomes were not used to determine eligibility to minimize selection bias by the sole author. However, the author decided that the symptoms of insomnia (difficulties initiating or maintaining sleep or affecting quality of life) would guide the clinically relevant outcomes of this SR. Outcome domains: (1) sleep quality, measured by sleep questionnaires and (2) sleep quantity, measured by sleep parameters. A third outcome domain of (3) presence of adverse events was chosen.

Studies combining magnesium supplementation with another intervention (co-intervention) were excluded. Non-English studies were excluded due to resource constraints.

### Data extraction

The authors JM and TP extracted the following data using a modified version of Cochrane’s (2020) template data collection form for intervention reviews of RCTs [15]: (1) General information (location, ethics, funding sources, conflicts of interest); (2) Study methods (aim, design of RCT, start/end dates, methods of randomization, allocation concealment and blinding); (3) Participants (inclusion/exclusion criteria, setting, method of recruitment, number per group, characteristics including age and sex, comorbidities, method of diagnosis); (4) Intervention and comparison (full description of magnesium supplementation regimen, duration of treatment, full description of control treatment, washout period for cross-over RCTs), (5) Follow-up (length, withdrawal rate and reasons); (5) Outcome data (description, definitions, time points measured/reported, measurement tools); (6) Results (effect estimates and precision per group, subgroups); (7): Analysis data (intention-to-treat or per-protocol analysis, comparability at baseline, statistical techniques); (7) key author conclusions. Discordance in data collection were adjudicated and consensus was reached for each discordant item.

### Assessment of risk of Bias

The authors carried out risk of bias assessments for included studies using the RoB 2.0 tools for individually randomized parallel-group and cross-over trials and included the following bias domains: randomization/allocation process, deviation from intended intervention, missing outcome data, outcome measurement and selective outcome reporting [16, 17]. Visualization of RoB 2.0 was produced using robvis [18]. Similarly, discordance was dealt with by adjudication amongst the authors until consensus was reached by following the appropriate algorithms.

### Data synthesis

Measures of effect were summarised using mean differences (post-intervention or change-from-baseline where appropriate) with standard deviations for continuous outcomes (no outcomes were dichotomized). When interventions were similar, and outcome measures were reported in the same scale, a meta-analysis was used to synthesise results and increase precision using random-effects model allowing for heterogeneity in the included studies. Heterogeneity was assessed qualitatively (by comparing studies for variability amongst populations, interventions, or designs) and statistically (with the chi-squared test and I^2^ statistic). Sensitivity analyses and funnel plots were not conducted due to insufficient number of studies. If a meta-analysis was not appropriate, and outcome data was not consistent across studies, vote counting based on direction of effect (not statistical significance) was employed as a synthesis method to complement the narrative synthesis. A modified effects direction plot was used to visualize vote counting for outcome domains, accounting for sample size and study quality [19, 20]. Quality of evidence across outcomes was assessed using the Grading of Recommendations, Assessment, Development and Evaluation (GRADE) approach, which incorporates the RoB 2.0 tool [21]. GRADE defines the certainty of evidence as the extent to which we can be confident that our results are representative of the true value of interest [21, 22]. This systematic review protocol was not registered, but follows PRISMA guidelines [23].

## Results

### Descriptions of studies

**Fig. 2:**
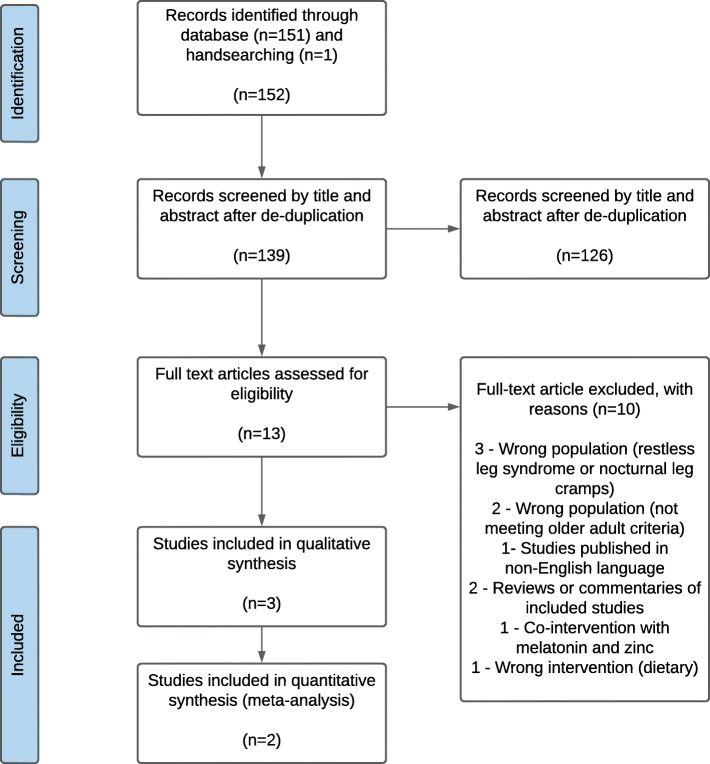
PRISMA flow diagram showing numbers of records identified at different phases of the systematic review

The search retrieved 152 records of which 13 were duplicates. After screening of titles and abstracts, 126 records were excluded, and 13 full text articles were obtained (see Fig. 2). The characteristics of the three studies included in the review are summarised Table 1. Across all studies, there were a total of 151 older adults from three countries, mostly without co-morbidities as no studies recruited participants with sleep-related breathing disorders or movement disorders. Daily elemental magnesium intake ranged from 320 mg to 729 mg taken two to three times per day using two formulations (magnesium oxide and magnesium citrate tablets). All interventions were compared to placebo. Duration of follow-up for outcome assessment ranged from 20 days to 8 weeks. Two validated sleep questionnaires were reported at baseline for insomnia diagnoses as well as follow-up outcomes: the Insomnia Severity Index (ISI) [24] and the Pittsburgh Sleep Quality Index (PSQI) [26]. One study [25] employed sleep electroencephalogram (EEG) to measure insomnia and another study had participants keep a detailed sleep diary [24].

**Table 1:** Characteristics of included studies

Study (Ref #)	Country	Methods (Design)	Participants (Mean Age) (Diagnosis Method)	Participant Inclusion Characteristics (Exclusion Criteria)	Intervention ^**a**^ (Duration)	Compari-son	Outcomes ^**b**^	Adverse Effects Reported?
Abbasi et al. 2012 [24]	Iran	RCT (Parallel)	46 elderly volunteers (65) (ISI)	Adults 60–75 years old with insomnia according to ISI and sleep-log questionnaires (BMI < 25 or > 34.9, dietary intake Mg > 75% RDA, serum Mg > 0.95 mmol/L, use of loop diuretics, cyclosporine, digoxin, amphotericin, hormonal treatment, renal disease, heart failure or sleep related movement or respiratory disease, psychiatric disorder, substance abuse, major life stressor, trans meridian flight in last 6 weeks)	500 mg elemental Mg daily Administered as 414 mg MgO PO BID (8 weeks)	Placebo	- ISI * - Sleep log * - Physical activity log - Food diary - Blood samples (Mg, cortisol, renin, melatonin)	No
Held et al. 2002 [25]	Germany	RCT (Cross-over)	12 healthy volunteers (69) (Sleep EEG)	Healthy older adults aged 60–80 years (Psychiatric disorders, cognitive impairment, recent stressful event, substance abuse, trans meridian flight in last 3 months, shift work, medical illness, aberrations in blood chemistry/EEG/ECG, sleep related respiratory or movement disorder)	729 mg elemental Mg daily Administered as an up-titration of 403 mg MgO PO daily × 3 days, 403 mg MgO BID × 3 days, and 403 mg MgO PO TID × 14 days (Treatment intervals of 20 days duration separated by 2 weeks washout)	Placebo	- Sleep EEG * - Blood samples (ACTH, cortisol, renin, AVP, ATII, aldosterone) ^c^	Yes (voluntary report)
Nielsen et al. 2010 [26]	United States of America	RCT (parallel)	100 older adults (59) (PSQI)	Adults > 51 years with global PSQI score > 5 indicating poor sleep quality (BMI > 40, respiratory tract disease, COPD, use of O2 or CPAP, use of ACEi, Mg-retaining or potassium sparing drugs)	320 mg elemental Mg daily Administered as 320 mg Mg citrate PO two tablets each morning and evening and one tablet at noon (8 weeks)	Placebo	- PSQI * - Food diary - Blood samples (Mg, erythrocyte Mg, calcium) - Urine samples (Mg, calcium, citrate)	No

a – Acronyms: *Mg* Magnesium; *PO* Per os / to be taken by mouth; *BID* Bis in die / twice a day; *TID* Ter in die / three times a day

b - All outcomes reported in each study are listed. Relevant outcomes to review question are starred (*)

c – Other acronyms: *ACEi* Angiotension Converting Enzyme inhibitors, *ACTH* Adrenocorticotropic hormone; *AVP* Arginine vasopressin; *ATII* Angiotension II, *BMI* Body mass index in kg/m^2^, *COPD* Chronic obstructive pulmonary disease, *CPAP* Continuous positive airway pressure, *ECG* Electrocardiogram, *EEG* Electroencephalogram, *RDA* Recommended daily amount

### Risk of Bias in included studies

**Fig. 3:**
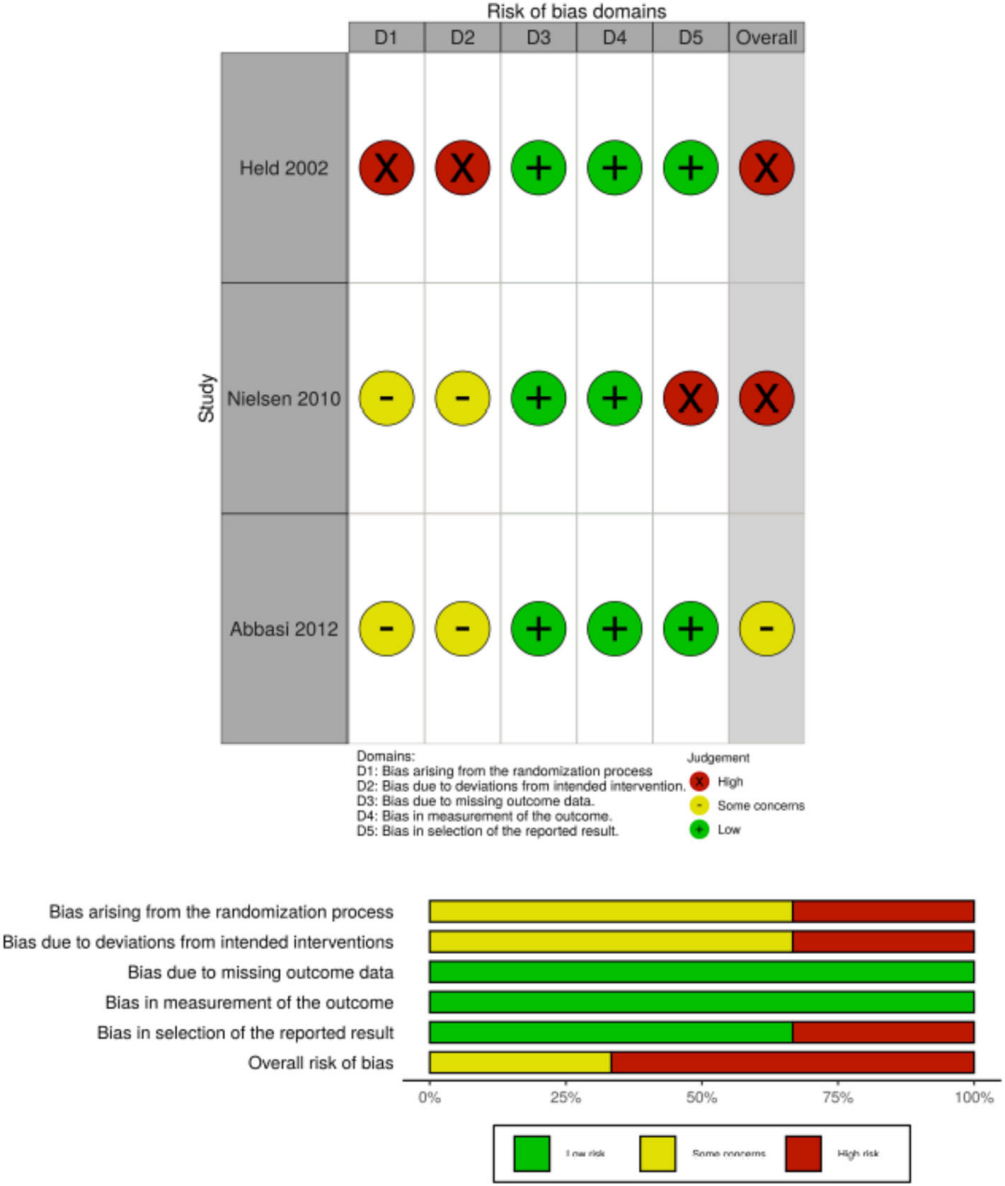
Risk of bias summary. N.B.: Author’s assessment per risk of bias domain for each included study (above) and author’s assessment per risk of bias domain by percentage across all included studies (below)

No study reported all elements required to make judgements in all risk of bias domains. While all studies were “double-blind” RCTs, zero studies reported methods of randomization sequence, allocation concealment or blinding; this placed all studies at some concern for bias, but Held (2002) was deemed higher risk for failing to report baseline characteristics [25]. Nielsen (2010) was deemed high risk for selective reporting because there was no analysis plan in the methods to explain reporting an effect of treatment on only one PSQI sub-score (out of seven) with an arbitrary cut off of ≤1 or > 1 [26]. Overall, Fig. 3 shows that two studies were assessed to be of high risk of bias (Held, 2002; Nielsen, 2010) and one was at some risk of bias (Abbasi, 2012).

### Effect of intervention

From the summary of findings table (Table 2), across all studies, the outcome domain with the greatest number of outcome measures is sleep parameters; followed by sleep questionnaires. There was no data available for adverse events. Overall, the authors have limited confidence in the effect estimates; that is to say, for each outcome, the true effect may be substantially different from the estimates of effect pooled or reported.

**Table 2:** Summary of Findings

Oral magnesium supplementation for older adults with insomnia							
Population: Older adults ≥55 years old with insomnia Intervention: Oral magnesium supplementation Comparison: Placebo							
Outcome (Duration of Follow Up)	No of Particip-ants (Studies)	Absolute Effects (Mean Difference ^**a**^ ± Standard Deviation – unless otherwise specified with *)		Relati-ve Effects ^b^	Quality of Evidence	Vote Count by Direction of Effect	Comments
		Placebo	Magnesium Supplementation				
Sleep Parameters							
Total sleep time (TST) Time from sleep onset to offset (min) (20 days to 8 weeks)	55 (2)	*The mean TST post-intervention ranged from 326.2 to 456.0 min	*The mean post-intervention TST in the intervention group was 16.06 min **higher** (95% CI: − 5.99 to 38.12; *p* = 0.15)	–	Low ^1,2^	Positive Effect ^i^	
Sleep onset latency (SOL) Time from wakefulness to initiation of sleep (min) (20 days to 8 weeks)	55 (2)	*The mean SOL post-intervention ranged from 34.7 to 84.0 min	*The mean post-intervention SOL in the intervention group was − 17.36 min **lower** (95% CI: − 27.27 to − 7.44, *p* = 0.0006)	–	Low ^1,2^	Positive Effect	Lower numbers indicate less night-time wakefulness and better insomnia symptomology of sleep initiation
Sleep efficiency (SE) Sum of REM & non REM sleep / total time in bed (h) (8 weeks)	43 (1)	MD = − 0.00 ± 0.05	MD = − 0.06 ± 0.01 h	–	Low ^3^	Positive Effect	
Early morning awakening (EMA) Premature termination of sleep (h) (8 weeks)	43 (1)	MD = 1.03 ± 0.02	MD = 1.01 ± 0.05	–	Low ^3^	Null Effect	Lower numbers indicate less early morning awakenings and better insomnia symptomology of sleep maintenance
Slow wave sleep (SWS) NREM stage 3 and 4 sleep (min) (20 days)	12 (1)	MD = + 10.1 ± 15.4	MD = + 16.5 ± 20.4	–	Very Low ^1,2,4^	Positive Effect	SWS, or deep sleep, is purported to be more restorative sleep.
Sleep Questionnaires							
Insomnia Severity Index Score from 0 to 28; ≥ 15 = clinical insomnia (8 weeks)	43 (1)	MD = − 0.5 ± 1.71	MD = − 2.38 ± 2.24	–	Low ^3^	Positive Effect	Lower scores indicate better sleep quality.
PSQI Score from 0 to 21; ≥ 5 = poor sleeper (8 weeks)	96 (1)	MD = − 4.1 See comment	MD = − 3.4 See comment	–	Low ^5^	Null Effect ^ii^	No numerical confidence intervals were reported but available in Figure form.
Adverse Events							
No data See comment	–	–	–	–	–	–	None of the studies reported adverse events

a – All mean differences (MD) are within group change from baseline mean differences unless otherwise specified with *. The * mean differences are between group post-intervention/treatment mean differences

b – No dichotomized outcomes were reported in any of the studies

Acronyms: *h* Hour; *min* Minute; *nREM* Non rapid eye movement; *REM* Rapid eye movement

**GRADE Working Group grades of evidence**

**High** certainty = very confident that the true effect lies close to that of the estimate of the effect

**Moderate** certainty = moderately confident that the true effect lies close to that of the estimate of the effect

**Low** certainty = limited confidence in the effect estimate, the true effect may be substantially different from the estimate of effect

**Very Low** certainty = very little confidence in the effect estimate, the true effect is likely to be substantially different from the estimate of effect

1 – Serious or concerning methodological limitations were detected in all studies, especially poor internal validity in the randomization process and bias arising from deviations from intended outcomes. Downgrade one level for risk of bias

2 – Only two studies included, with wide confidence intervals and total sample size of 55. Downgrade one level for imprecision

3 – Only one study included. Some concerns for risk of bias in the randomization process and bias arising from deviations from intended outcomes (same as above) mainly due to poor reporting. Downgraded one level. Total sample size of 43. Downgraded one level for imprecision. (Total: 2 levels downgraded)

4 –SWS is a surrogate outcome for insomnia symptoms, the main outcome assessed in the review question. While there is biological plausibility that SWS may help with restorative sleep, there is limited evidence in SWS to improvement in insomnia symptoms. Downgraded one level for indirectness of evidence

5 – Only one study included. High risk of bias from selective reporting. Downgrade one level. Total sample size of 96. Downgraded one level for imprecision

**Voting by Direction of Effect**

i – Despite lack of statistical significance in the meta-analysis, vote counting was conducted purely by observed direction of effect alone

ii – Each question of the PSQI is scored 0, 1,2 or 3. Thus, a difference of less than 1 is categorized as a null effect

Reference: Schünemann HJ, Higgins JPT, Vist GE, Glasziou P, Akl EA, Skoetz N, Guyatt GH. 2019. Chapter 14: completing ‘summary of findings’ tables and grading the certainty of the evidence. In: Higgins JPT, Thomas J, Chandler J, Cumpston M, Li T, Page MJ, Welch VA (editors). Cochrane handbook for systematic reviews of interventions version 6.0 (updated July 2019). Cochrane. Available from

www.training.cochrane.org/handbook (http://www.training.cochrane.org/handbook)

**Fig. 4:**
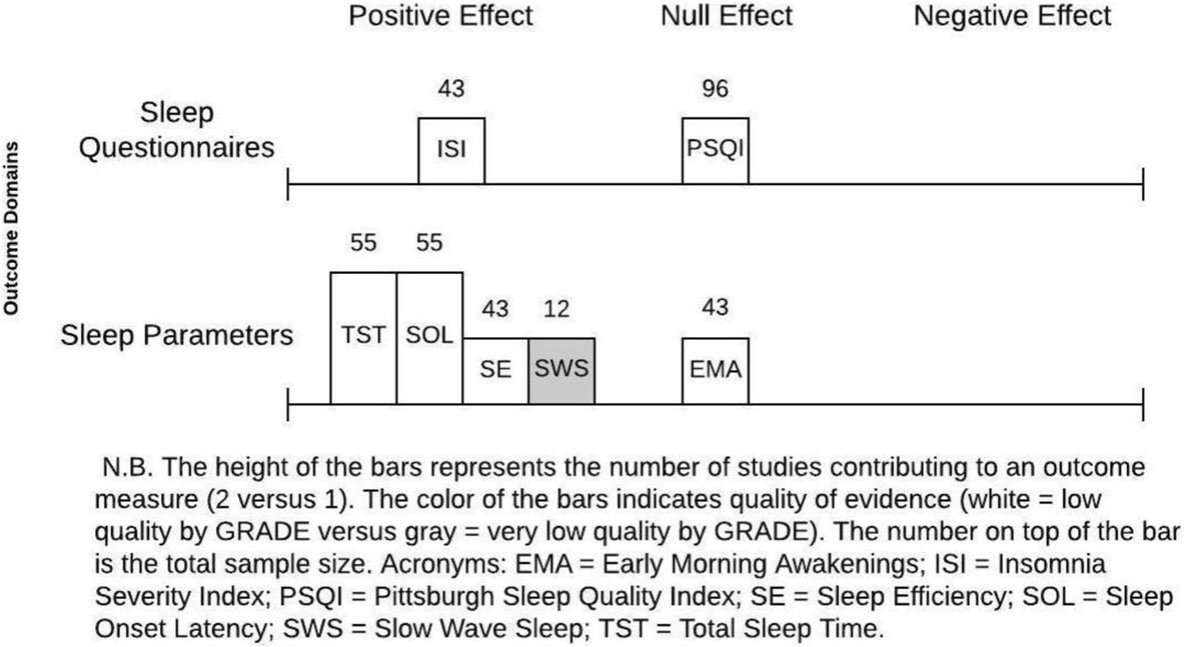
Evidence by vote count for change in insomnia outcomes after magnesium supplementation, a modified effect-direction plot

In the face of these limitations, it is difficult to comment on the significance of an effect estimate for insomnia symptoms by magnitude or precision. Instead, Fig. 4 helps to answer the question of “does magnesium supplementation have any evidence of effect”? Across all outcomes, by frequency alone, there was a positive effect of magnesium supplementation on improvement of sleep parameters. For sleep questionnaires, one study [24] showed improvement with some concerns of risk of bias and one study showed null effect with a greater sample size but higher risk of bias [26].

#### Sleep parameters

**Fig. 5:**
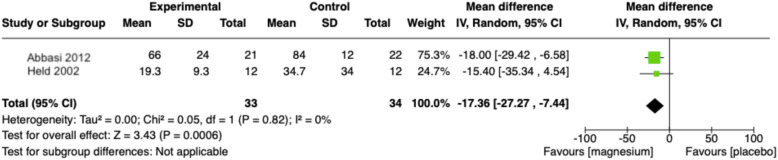
Forest plot comparison of magnesium supplementation compared to placebo for sleep onset latency (SOL) outcome. N.B.: Inverse-variance weight method applied; Sleep onset latency converted to same unit (minutes); Statistical tests for heterogeneity (Chi^2^ and I^2^) can be unreliable with small sample sizes – heterogeneity explored using other strategies

**Fig. 6:**
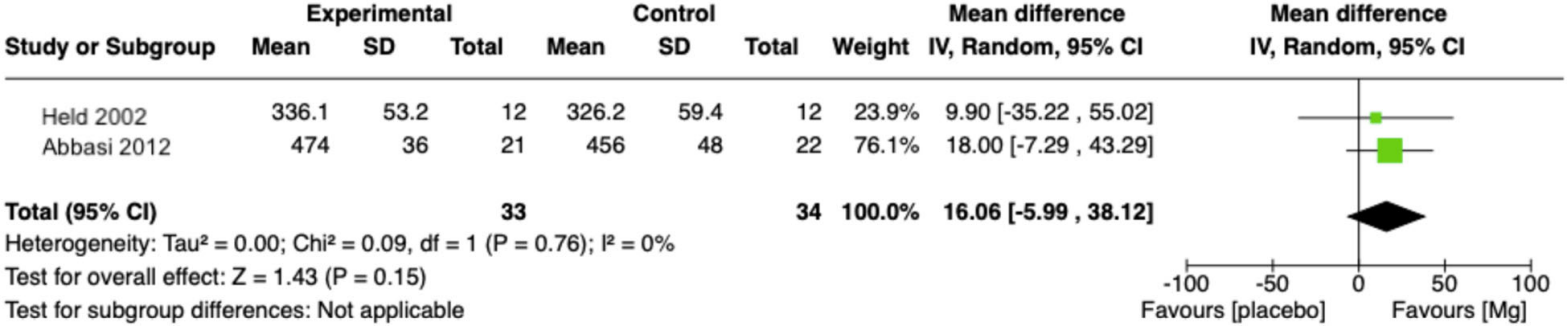
Forest plot comparison of magnesium supplementation compared to placebo for total sleep time (TST) outcome N.B.: Inverse-variance weight method applied; Sleep onset latency converted to same unit (minutes); Statistical tests for heterogeneity (Chi^2^ and I^2^) can be unreliable with small sample sizes – heterogeneity explored using other strategies

There is minimal evidence of positive effect, of low to very low certainty, to suggest that magnesium supplementation improves insomnia symptoms as measured through sleep parameters. Two studies collected information on various sleep times [24, 25]. The pooled results of these trials showed that the post-intervention sleep onset latency (SOL) time was 17.36 min less after magnesium supplementation compared to placebo (95% CI − 27.27 to − 7.44, *p* = 0.0006) (Figs. 5 and 6). The pooled results for total sleep time (TST) was 16.06 min higher in the magnesium supplementation group but statistically insignificant (95% CI: − 5.99 to 38.12; *p* = 0.15). Both analyses had low statistical evidence of heterogeneity between trials (SOL: I^2^ = 0, chi2 test *p* = 0.05; TST: I^2^ = 0, chi^2^ test *p* = 0.09) but Table 1 suggests that there was heterogeneity in the methods of measurement (sleep log versus sleep EEG) and design of RCT (parallel versus cross-over). One study further reported that magnesium supplementation brought about statistically significant improvements in SE compared to placebo but not improvement in EMA. A study of poorer internal validity reported that slow wave sleep time improved in the magnesium group compared with placebo [25].

#### Sleep questionnaires

There is indeterminate evidence of effect direction, of low certainty, to suggest that magnesium supplementation improves insomnia symptoms as measured through validated questionnaires. Abbasi (2012) showed a greater improvement in ISI score in the intervention group compared to placebo but Nielsen (2010) showed equal improvements in PSQI score in both groups.

### Adverse events

Held (2002) reported that all participants had soft stools, a known (and often desirable) side effect of oral magnesium. The remaining studies did not report any unintended adverse effects.

## Discussion

### Summary of results & explanation of findings

This SR aimed to assess whether oral magnesium supplementation was effective and safe for insomnia in older adults. The findings suggest that the true effect of magnesium supplementation on insomnia symptoms lies somewhere between a positive effect and a null effect in comparison to placebo as measured by sleep parameters and questionnaires. The clinical significance of these findings, such as an improved sleep onset latency time of 17.36 min is debateable.

These findings do not give a clear answer which is consistent with the mixed literature showing uncertain association between dietary magnesium intake and sleep symptoms [4, 6]. This SR adds to the growing body of literature that highlights that there is insufficient evidence, or only evidence of low quality, to make recommendations on commercially available CAM supplements with regards to their effectiveness or safety. However, these findings do show a possible positive effect in sleep parameters and did not identify any adverse effects in doses of less than 1 g of elemental magnesium per day in twice or three times a day dosing (existing literature suggests doses greater than 5 mg have been associated with renal and cardiac side effects). Given that oral magnesium is very cheap and widely available, the authors can found no evidence preventing the recommendation of oral magnesium supplementation for insomnia symptoms in older adults. To definitely answer the research question posed, more rigorous studies are required in this area, especially in the context of increasing prevalence of CAMs and their use in conjunction with other treatments requiring better evidence for physicians to make recommendations either for or against such supplementation [5].

### Limitations & future directions

This review looked at oral magnesium by examining the gold standard evidence of RCTs to answer a question of medication effectiveness. However, the critical limitation of this SR is the overall low quality of evidence and high risk of bias in all included studies. Instead, a revised search of non-RCTs may offer evidence of higher certainty and be more conducive to the part of the research question exploring adverse effects. Searches of supplementary databases (e.g. regulatory sources) may include on-going or unpublished trials,; for example, there is one on-going trial examining magnesium compared with potassium supplementation for sleep in patients with diabetes from clinicaltrials.gov. Furthermore, future reviews may consider looking beyond RCTs to include high quality observational studies. With more studies, construction of a funnel plot would help to answer the question of publication bias with CAM therapies. Allowing for studies in all languages may reduce selection bias, as magnesium supplementation may be more prevalent in non-English speaking countries.

## Conclusion

This review confirms that the quality of literature is substandard for physicians to make well-informed recommendations on usage of oral magnesium for older adults with insomnia. However, given that oral magnesium is very cheap and widely available, RCT evidence may support oral magnesium supplements (less than 1 g quantities given up to three times a day) supplementation for insomnia symptoms.

## Supplementary Information

**Additional file 1.** Summary of Search Strategy Across all Databases Including Key Concepts, Key Words, MESH terms and Record Numbers. Provides additional details of the review search strategy.

**Additional file 2.** EMBASE Unedited and Exported Database Search Strategy Including the Validated Randomized Control Trial Filter. Exported review search strategy.

## Data Availability

Not applicable to this article as no datasets were generated or analysed.

## Abbreviations

AMED: Allied and complementary medicine
CAM: Complementary and alternative medicine
EEG: Electroencephalogram
GRADE: Grading of recommendations assessment, development and evaluation
ISI: Insomnia severity index
MESH: Medical indexed standardized subject terms
Mg: Magnesium
OTC: Over-the-counter
PSQI: Pittsburgh sleep quality index
RCT: Randomized control trial
RoB: Risk of bias
SOL: Sleep onset latency
SR: Systematic review
TST: Total sleep time
