# Correction: Oral magnesium supplementation for insomnia in older adults: a Systematic Review & Meta-Analysis

**DOI:** 10.1186/s12906-024-04721-w

**Published:** 2024-12-19

**Authors:** Jasmine Mah, Tyler Pitre

**Affiliations:** 1 Department of Medicine, Dalhousie University, Halifax, NS Canada; 2 Division of Internal Medicine, McMaster University, Hamilton, ON Canada; 3 Michael G. DeGroote School of Medicine (Waterloo Regional Campus), McMaster University, Hamilton, ON Canada

**Correction: BMC Complement Med Ther 21, 125 (2021)**

10.1186/s12906-021-03297-z

Following publication of the original article [1], the authors reported an error in text.

- In Abbreviation section, the word “MeSH” should be changes to “MESH”.
- lines 376–379 would read much better if “forimproved sleep parameters after magnesiumsupplementation” were deleted.
- they update the first paragraph in results section to match PRISMA 141 records -->152 records, 12 duplicates --> 13 duplicates, 120 records were excluded --> 126 records were excluded.
- Line 122–123: CENTRAL will not find reviews, but contains RCTs. Cochrane CENTRAL --> Cochrane Database of Systematic Reviews (CDSR).

The original article has been corrected.
