# Supplementary material for: Oral magnesium supplementation for insomnia in older adults: a Systematic Review & Meta-Analysis
Source: BMC Complement Med Ther. 2021 Apr 17;21:125. doi: 10.1186/s12906-021-03297-z (PMC8053283; doi:10.1186/s12906-021-03297-z)
Supplement: Supplementary file 1 — Additional file 1. Summary of Search Strategy Across all Databases Including Key Concepts, Key Words, MESH terms and Record Numbers. Provides additional details of the review search strategy. [file 12906_2021_3297_MOESM1_ESM.docx]

**Additional File 1:**

**Summary of Search Strategy Across all Databases Including Key Concepts, Key Words, MESH terms and Record Numbers**

| **Database** | **Medline** | **Embase Classic + Embase ^a^** | **Allied and Complementary Medicine (AMED)** | **https://www.clinicaltrials.gov/** |
| --- | --- | --- | --- | --- |
| **Platform ^b^** | Ovid | Ovid | Ovid | N/A |
|  |  |  |  |  |
| **Rationale for Inclusion ^c^** | ​C​overs all aspects of clinical medicine, biomedicine, nursing, dentistry, allied health, health policy, genetic, etc. Also suggested by LSHTM librarian. | Comprehensive pharmacological and biomedical database renowned for extensive indexing of drug information | Contains references to articles on allied and alternative medicine. Many of the journals covered are not indexed by any other biomedical sources. | Contains public and privately funded clinical trials, often including trials for drugs applying for a FDA Investigational new Drug Application |
|  |  |  |  |  |
|  |  |  |  |  |
| **Search Last Updated** | October18, 2020 | October 18, 2020 | October 12, 2020 | March 15, 2021 |
|  |  |  |  |  |
| **Database Timespan** | 1946 to October Week 1 2020 | 1947 to October 16, 2020 | 1985 to October 2020 | September 2008 Onwards |
|  | **Concept 1: Insomnia** | | | |
| **Key Words (# of records)** | **1.** (Transient or acute or chronic or age-induced or geriatric* or elder* or primary or secondary) adj2 insomnia).mp | | | **1.** Insomnia |
|  | (1914) | (4014) | (68) |  |
|  | **2.** (earl* adj2 awak*).mp | | |  |
|  | (591) | (1210) | (9) |  |
|  | **3.** (Sleep adj3 (deprivation or quality or onset or hygiene or stage or med* or problem* or disorder* or inability* or difficult* or maintain* or initiat*)).mp | | |  |
|  | (72951) | (180046) | (1424) |  |
|  | **4.** (Insomnia* or sleep* or wake*).mp | | |  |
|  | (195162) | (180046) | (3514) |  |
| **MESH ^c^ (# of records)** | **5.** exp "Sleep Initiation and Maintenance Disorders"/ (12878) | **5.** exp insomnia/ or exp primary insomnia/ (68212) | **5.** exp Insomnia/ (306) |  |
| **Combination (# of records)** | **6.** 1 or 2 or 3 or 4 or 5 (195249) | **6.** 1 or 2 or 3 or 4 or 5 (401928) | **6.** 1 or 2 or 3 or 4 or 5 (3515) |  |
|  | **Concept 2: Older Adults** | | | |
| **Key Words (# of records)** | **7.** (Elder* or senior*).mp. | | |  |
|  | (263489) | (614309) | (7153) |  |
|  | **8.** ((Longterm or long-term or nursing) and care).mp | | |  |
|  | (331169) | (207078) | (6063) |  |
|  | **9.** ((Nursing or care) adj2 home).mp | | |  |
|  | (75766) | (131379) | (4400) |  |
|  | **10.** (Sexagenarian* or Septuagenarian* or Octogenarian* or Nonagenarian* or Centenarian* or Supercentenarian*).mp | | |  |
|  | (5475) | (9585) | (44) |  |
|  | **11.** ((old or older) adj1 age).mp | | |  |
|  | (62598) | (109157) | (1015) |  |
|  | **12.** (Ageing or aging or aged or frail* or geriatri* or geronto* or psychoger* or geropsych* or "late* life*" or "late* adulthood" or "old* adult*" or "old* age*" or "old* people*" or "old* person*" or "old* citizen*" or "old* men" or "old* women" or "old* male*" or "old* female*" or "old* patient*" or "old* population*" or "old old" or "very old" or "senior citizen*" or pensioner* or retired or retirement).mp | | |  |
|  | (5505601) | (5370157) | (33578) |  |
|  | **13.** ("55 years" or "60 years" or "64 years" or "65 years" or "70 years" or "75 years" or "79 years" or "80 years" or "85 years" or "90 years" or "95 years" or "older than 55" or "older than 60" or "older than 65" or "older than 70" or "older than 75" or "older than 80" or "older than 85" or "older than 90" or "older than 95").mp | | |  |
|  | (253841) | (471455) | (3559) |  |
| **MESH (# of records)** | **14.** exp "Aged, 80 and over"/ or exp Aged/ (3062714) | **14.** aged/ or aging/ or elderly care/ (3282484) | **14**. exp Aged/ (15853) |  |
|  | **15.** exp Geriatrics/ (29693) | **15.** exp geriatrics/ (45038) | **15.** exp Geriatrics/ or exp Nursing homes/ or exp Aging/ (4864) |  |
|  | **16.** exp Geriatric Psychiatry/ (2338) | **16.** exp gerontopsychiatry/ (7649) | **16.** exp Geriatric nursing/ or exp Geriatric assessment/ (604) |  |
| **Combination (# of records)** | **17.** 7 or 8 or 9 or 10 or 11 or 12 or 13 or 14 or 15 or 16 (5806728) | **17.** 7 or 8 or 9 or 10 or 11 or 12 or 13 or 14 or 15 or 16 (5790346) | **17.** 7 or 8 or 9 or 10 or 11 or 12 or 13 or 14 or 15 or 16 (43527) |  |
|  | **Concept 3: Oral Magnesium Supplementation ^d^** | | | |
| **Key Words or MESH (# of records)** | **18.** exp Magnesium Silicates/ or exp Magnesium Deficiency/ or exp Magnesium Sulfate/ or magnesium.mp. or exp Magnesium Compounds/ or exp Potassium Magnesium Aspartate/ or exp Magnesium Hydroxide/ or exp Magnesium Chloride/ or exp Magnesium/ or exp Magnesium Oxide/ (101705) | **18.** magnesium oxide/ or magnesium carbonate/ or magnesium citrate/ or magnesium chloride/ or magnesium sulfate/ or magnesium hydroxide/ or magnesium/ or magnesium.mp. (174363) | **18.** magnesium.mp. or exp Magnesium/ (290) | **2.** magnesium |
| **Combination (# of records)** | **19**. 6 and 17 and 18 (89) | **19.** 6 and 17 and 18 (289) | **19.** 6 and 17 and 18 (1) | **3.** 1 and 2 (11) |
| **Record Number (#) After RCT Filter Applied** | | | | |
|  | 37 | 102 | 1  No filter applied ^e^ | N/A |
| Total Number of Records Identified Through Database Searching = 37 + 102 + 1 + 11 = **151** | | | | |

a – Embase Classic included to expand search allowing for data dating back to 1947

b – OVID as a platform was used to access all three databases. This allowed for all the key word searches (& their syntaxes) to be the same in Medline, Embase & AMED.

c – Acronyms in alphabetical order: MESH = MEdical Subject Heading, RCT = randomized controlled trial (see Additional File 2 for details of this filter)

d – Magnesium was the most specific concept used in the search strategy. The key word magnesium was therefore used instead of key words such as oral magnesium or magnesium supplementation to broaden the search (especially in a smaller database such as AMED or clinicaltrials.gov).

e– AMED & clinicaltrials.gov are smaller databases; hence the RCT filter was not necessary.

**N.B. To search for previous reviews on this topic, a validated search filter for systematic reviews and meta-analyses (Scottish Intercollegiate Guidelines Network, 2019) was applied in place of the RCT filter in MEDLINE and EMBASE.**
