# Supplementary material for: Oral magnesium supplementation for insomnia in older adults: a Systematic Review & Meta-Analysis
Source: BMC Complement Med Ther. 2021 Apr 17;21:125. doi: 10.1186/s12906-021-03297-z (PMC8053283; doi:10.1186/s12906-021-03297-z)
Supplement: Supplementary file 2 — Additional file 2. EMBASE Unedited and Exported Database Search Strategy Including the Validated Randomized Control Trial Filter. Exported review search strategy. [file 12906_2021_3297_MOESM2_ESM.docx]

**Additional File 2:**

**EMBASE Unedited and Exported Database Search Strategy Including the Validated Randomized Control Trial Filter**

Database: Embase Classic+Embase <1947 to 2020 October 16>

Search Strategy:

--------------------------------------------------------------------------------

1 ((Transient or acute or chronic or age-induced or geriatric* or elder* or primary or secondary) adj2 insomnia).mp. (4014)

2 (earl* adj2 awak*).mp. (1210)

3 (Sleep adj3 (deprivation or quality or onset or hygiene or stage or med* or problem* or disorder* or inability* or difficult* or maintain* or initiat*)).mp. (180046)

4 (Insomnia* or sleep* or wake*).mp. (401775)

5 exp insomnia/ or exp primary insomnia/ (68212)

6 1 or 2 or 3 or 4 or 5 (401928)

7 (Elder* or senior*).mp. (614309)

8 ((Longterm or long-term or nursing) adj2 care).mp (207078)

9 ((Nursing or care) adj2 home).mp. [ (131379)

10 (Sexagenarian* or Septuagenarian* or Octogenarian* or Nonagenarian* or Centenarian* or Supercentenarian*).mp. (9585)

11 ((old or older) adj1 age).mp. (109157)

12 (Ageing or aging or aged or frail* or geriatri* or geronto* or psychoger* or geropsych* or "late* life*" or "late* adulthood" or "old* adult*" or "old* age*" or "old* people*" or "old* person*" or "old* citizen*" or "old* men" or "old* women" or "old* male*" or "old* female*" or "old* patient*" or "old* population*" or "old old" or "very old" or "senior citizen*" or pensioner* or retired or retirement).mp. (5370157)

13 ("55 years" or "60 years" or "64 years" or "65 years" or "70 years" or "75 years" or "79 years" or "80 years" or "85 years" or "90 years" or "95 years" or "older than 55" or "older than 60" or "older than 65" or "older than 70" or "older than 75" or "older than 80" or "older than 85" or "older than 90" or "older than 95").mp. (471455)

14 aged/ or aging/ or elderly care/ (3282484)

15 exp geriatrics/ (45038)

16 exp gerontopsychiatry/ (7649)

17 7 or 8 or 9 or 10 or 11 or 12 or 13 or 14 or 15 or 16 (5790346)

18 magnesium oxide/ or magnesium carbonate/ or magnesium citrate/ or magnesium chloride/ or magnesium sulfate/ or magnesium hydroxide/ or magnesium/ or magnesium.mp. (174363)

19 6 and 17 and 18 (289)

20 clinical trial/ (988497)

21 randomized controlled trial/ (596939)

22 controlled clinical trial/ (463980)

23 multicenter study/ (245363)

24 phase 3 clinical trial/ (45696)

25 phase 4 clinical trial/ (3798)

26 exp randomization/ (86726)

27 single blind procedure/ (38280)

28 double blind procedure/ (173000)

29 crossover procedure/ (62819)

30 placebo/ (358080)

31 randomi?ed controlled trial$.tw. (223285)

32 rct.tw. (36191)

33 (random$ adj2 allocat$).tw. (42486)

34 single blind$.tw. (24645)

35 double blind$.tw. (212524)

36 ((treble or triple) adj blind$).tw. (1163)

37 placebo$.tw. (309653)

38 prospective study/ (589450)

39 or/20-38 (2313338)

40 Case Study/ (76831)

41 case report.tw. (432770)

42 abstract report/ or letter/ (1134472)

43 Conference proceeding.pt. (0)

44 Conference abstract.pt. (3724845)

45 Editorial.pt. (645303)

46 Letter.pt. (1104556)

47 Note.pt. (788408)

48 or/40-47 (6739479)

49 39 not 48 (1729304)

50 19 and 49 (102)

***************************

Notes on Search Abbreviations:

- mp = to search: title, abstract, original title, name of substance word, subject heading word, floating sub-heading word, keyword heading word, organism supplementary concept word, protocol supplementary concept word, rare disease supplementary concept word, unique identifier, synonyms
- tw = to search: title, abstract
- pt = to search: publication type
- exp = exploded medical subject heading (MESH)

Lines 20-49 are from the SIGN RCT search filter.
